# Indicators and Dimensions of Clients’ Satisfaction With Veterinary Services Delivery Performance in a Companion Animal Hospital in Accra, Ghana

**DOI:** 10.1002/vms3.70331

**Published:** 2025-04-10

**Authors:** Paa Kobina Turkson, Celine Naa Dede Coppson, Anthony Joe Turkson

**Affiliations:** ^1^ School of Veterinary Medicine University of Ghana Legon Ghana; ^2^ Department of Mathematics, Statistics and Actuarial Science Takoradi Technical University Takoradi Ghana

**Keywords:** client satisfaction, companion animals, dimensions, Ghana, indicators, veterinary services

## Abstract

**Background:**

Client satisfaction surveys present a chance to find out which services offered by a provider meet or exceed the expectations of clients, and therefore should be maintained, while services that fall short of expectations are identified for improvement.

**Objectives:**

The aims were to determine if correlations existed between dimensions of service quality (tangibles, reliability, responsiveness, assurance and empathy) and client satisfaction, find out which of 15 selected indicators of service delivery performance were significantly associated with client satisfaction and serve as predictors for satisfaction and determine client satisfaction indices for services rendered at a companion animal hospital.

**Methods:**

A questionnaire, covering background information and indicators and dimensions of satisfaction, was administered to 208 respondents. Data analyses involved proportions, mean scores, Cronbach reliability test, relative importance indices, correlations, multiple regressions and client satisfaction indices.

**Results:**

For indicators, the prediction equation was as follows: Overall satisfaction = −44.1 + 15.3 × Drugs availability. For dimensions, the equation was as follows: Overall satisfaction (mean score) = 2.39 + 0.19 × Reliability mean score. The customer satisfaction score was 97.5%, and the mean satisfaction rate was 84.3%. The composite customer satisfaction score was also 84.3%. The customer satisfaction index was 81.5%.

**Conclusion:**

The study provides, for the first time, attributes considered by clients as contributing to satisfaction with veterinary services delivery for pets in Accra, Ghana. Regression analyses revealed that for dimensions, the reliability mean score, and for indicators, the availability of drugs were the main predictors of overall satisfaction with services delivery. Client satisfaction indices were very high.

## Graphical abstract

The study reported on satisfaction indices and prediction models for dimensions and indicators of service quality for clients accessing services in a companion animal hospital in Ghana. In general, client satisfaction indices were very high indicating good delivery performance.

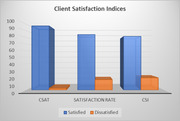

## 1 Introduction

Client satisfaction is critical if service providers are to attract new clients, retain existing clients and pursue and ensure clients’ loyalty (Rasyida et al. 2016). Client satisfaction may result in repeat use, loyalty and customer retention (Zairi 2000). Positive attitude towards a service is enhanced by customer satisfaction, resulting in a greater likelihood of further use (White 2010), while dissatisfaction elicits negative attitudes towards a brand and less likelihood of continued use (Zhou 2004).

A major factor identified as distinguishing between services and building competitive advantage in healthcare is service quality (Byju and Srinivasulu 2014). Poor quality of healthcare delivery by providers in health facilities may result in loss of clients, lives, revenue, material resources, morale, staff, recognition, trust and/or respect (Bannerman et al. 2002), leading to dissatisfaction and apathy towards services affecting effectiveness and efficiency (Turkson 2008/2009). Service quality had a positive effect on customer loyalty in veterinary clinics in San Miguel, Peru (Mathos 2023).

Satisfaction surveys have been used to collect clients’ opinions, experiences and needs for services. Client's perception of service quality needs to be identified to allow management to direct marketing efforts to ensure that expectations of clients are met (Rasyida et al. 2016). This entails identifying, prioritizing and improving weak service areas and ensuring that valuable resources are allocated to most effective areas. Satisfaction is, often, a measure of client's perception of quality (Moreau 2003). Client satisfaction is defined as the level of satisfaction experienced by clients who used a service, and it reflects the gap between the expected service and the experience of the service from the client's point of view (Smith and Engelbrecht 2001). Measuring client satisfaction can help in ensuring a more stable satisfied clientele in a practice (Moreau 2003). Further, highly satisfied clients tend to feel that they have received high‐quality service, while disappointment with the quality of services results in dissatisfied clients (Moreau 2003).

There are many ways of assessing client satisfaction. One is SERVPERF, which is a modification of the SERVQUAL instrument. SERVPERF is said to be effective in measuring the performance of service facilities, pointing out deficiencies needed to be improved (Chase et al. 2001). SERVPERF is reportedly a better scale than SERVQUAL (Babakus and Boller 1992; Cronin and Taylor 1992). It is more efficient and explains greater variance in overall service quality (S. K. Jain and Gupta 2004).

There is very little published information on satisfaction with delivery of pet veterinary services in Ghana. Previous work had reported on satisfaction with delivery of veterinary services to mainly livestock owners including poultry in peri‐urban areas in Ghana (Turkson 2008a, 2008b, 2009a, 2009b, 2010, 2011). This study fills in the gap by providing information on how clients perceive services rendered in a companion animal hospital in Accra, Ghana.

An objective of the study was to determine whether or not correlations existed between dimensions of service quality (tangibles, reliability, responsiveness, assurance and empathy) and client satisfaction and the extent and significance of such relationships. A second objective was to find out which of 15 selected indicators of service delivery performance were significantly associated with client satisfaction and could be used in predicting overall client satisfaction with services delivered. A third objective was to determine client satisfaction indices for services rendered at a companion animal hospital in Accra.

## 2 Methods

The study was done in the Small Animal Teaching Hospital in Accra, Ghana, used for clinical training of students in the School of Veterinary Medicine, University of Ghana. It is the largest veterinary teaching and referral hospital in Ghana. A questionnaire, similar to one used by Turkson (2008b) with slight modifications, was adopted. The targeted respondents were clients accessing services provided by the hospital. The inclusion criterion was clients who had attended the facility at least once before the date of the interview. The exclusion criterion was all new clients of the facility; that is, clients who were coming to the facility for the very first time on the day of encounter. The questionnaire administration, done by the second author only to ensure consistency, was from March to October 2023, but no data were collected in August 2023. The total number of eligible clients was 564, out of which 208 (36.9%) were interviewed.

An electronic version of the questionnaire in Google Forms was administered to respondents in person who agreed to be interviewed during hospital visits with their animals. Questionnaire administration took between 10 and 16 min (average 12 min). Data were exported to Microsoft Excel sheets and analyzed using SPSS version 25. Analyses involved proportions, percentages and mean scores (with standard deviations) and multiple regressions.

SERVPERF, a modification of SERVQUAL (a satisfaction survey instrument), was adopted for use (Byju and Srinivasulu 2014; Dassanayake and Weerasiri 2015; Rasyida et al. 2016). SERVPERF measures service quality. Following A. Jain (2012), reliability was defined as the ability to deliver promises on time; responsiveness was the degree to which customers perceived service provider's readiness to assist promptly, and assurance was the degree of courtesy of service provider's workers and ability to communicate trust to customers. Empathy was defined as care and importance given by the service provider to an individual customer, while tangibles was the evidence of facilities, personnel and communication materials used by the service provider in offering services to customers. The unweighted SERVPERF was calculated as indicated in Turkson (2008/2009).

Cronbach's reliability test was used to check whether or not the respondents’ scores on any item statement were related to their scores on the others (Rasyida et al. 2016). The Cronbach alpha test was done using SPSS version 25 software to measure reliability or internal consistency within the dimensions and indicators tested.

The Relative Importance Index (RII) is a statistical measure used to determine the relative importance or contribution of independent variables in predicting a dependent variable (Rangith n.d.). The RII is defined as a ‘non‐parametric technique used for analyzing structured questionnaire responses for data involving ordinal measurement of attitudes’ (Sakhare and Patil 2019). The RII was used to identify which dimensions or indicators were to be prioritized to achieve client satisfaction. The performance of the veterinary service provider on each of the attributes or indicators as assessed by the clients was derived by transforming the Likert scale scores into the RII calculated as ∑*W*/(*A* × *N*), where *W* was weighting assigned to each variable by respondent, *A* was the highest weight and *N* was total number of respondents (Nuvey et al. 2023). The higher the RII value, the more important was the criterion. The interpretation was as follows: High: 0.8<RII<1.0; High‐Medium: 0.6<RII<0.8; Medium: 0.4<RII<0.6; Medium‐Low: 0.2<RII<0.4 and Low: 0.0<RII<0.2 (Tarek et al. 2022). The RII was calculated in Excel.

To find the correlation between five dimensions of service quality (tangibles, reliability, responsiveness, assurance and empathy) and overall satisfaction, Spearman's rho test was used in SPSS. The test was also done with the following 15 indicators: affordability of services, staff attitude, staff technical competence, quality of services, staff efficiency, staff effectiveness, accessibility of services, needs met, total time spent in facility, timeliness, service availability, availability of pet vaccinations, availability of drugs, charges for services and cost of drugs. Cross tabulations were done for the dependent variable (Overall satisfaction) and the 15 independent variables. Binomial logistic regression was used in SPSS to find a predictive formula for the dependent variable from the independent variables. For this, the indicators were reclassified as follows: Service effectiveness/efficiency/quality/Staff attitude/competence: very poor/poor = 0; fair/good/very good = 1; Accessibility: very difficult/difficult = 0; fair/easy/very easy = 1; Timeliness: long/too long = 0; short/acceptable = 1; Service charges/Drug cost: expensive/very expensive = 0; fair/reasonable = 1; Service/drug/vaccination availability: unavailable/sometimes available = 0; available/always available = 1; Service affordability: unaffordable = 0; fairly affordable/affordable = 1; Meeting needs: poorly met/somehow met = 0; fairly met/met/very much met = 1; Total time spent: too long/long = 0; fair/reasonable = 1; Overall satisfaction: very dissatisfied/dissatisfied/somewhat satisfied = 0 and satisfied/very satisfied = 1. Differences between satisfied and dissatisfied respondents were tested for significance by χ^2^ test at *α* = 0.05. To check for collinearity, pairs of the independent indicators were cross tabulated, and odds ratios (ORs) were calculated in SPSS version 25. ORs above 10 were interpreted as showing strong collinearity; therefore, one of the pairs had to be dropped (Turkson 2011). Fishers’ exact test, Kappa statistic test and Mantel–Haenszel *χ* ^2^ test were used for statistical inferences.

Multiple regression analyses in stepwise mode were done in SPSS for the relationship between five independent dimensions of service quality and the dependent variable (overall satisfaction). The average perception score of each dimension was used. Similarly, stepwise Binomial logistic regressions were done for the dependent variable and the 15 independent indicators mentioned earlier.

Various satisfaction indices were calculated. Client satisfaction rate (%), based on overall satisfaction, used the formula {(Customer's score/highest possible score) × 100} (Taylor 2023). The mean was calculated and reported. Customer Satisfaction Score (CSAT) (%) was {(Total number of very satisfied or satisfied respondents/Total number of respondents) × 100}; and composite customer satisfaction score (proportion of satisfied customers) (%) = [(Sum of all scores/Total number of respondents) × 100] (Bishop 2024). Customer satisfaction index was calculated as [(Sum of each identified attribute/Total number of attributes) × 100] (Process Street n.d.). CSI interpretations were as follows: 0%–40% = Very dissatisfied—extreme customer dissatisfaction; 40%–60% = Dissatisfied—customer dissatisfaction; 60%–75% = Hesitation—some problems in customer satisfaction; 75%–90% = Satisfied—few problems in customer satisfaction and 90%–100% = very satisfied—customer satisfaction (Wolniack and Skotnicka, 2008).

### 2.1 Ethical Considerations

Informed verbal consent was obtained from each respondent before the questionnaire was administered. The questionnaire assured anonymity by not having any personal identifiers traceable to individuals apart from gender and age categories. Permission for the study was obtained from the coordinator of the companion animal hospital.

### 2.2 Limitations of the Study

No sampling frame was available, so purposive sampling technique was adopted which could have introduced some bias. Some clients did not participate because they were in a hurry to leave, thereby reducing the number interviewed. Clients attending only one veterinary facility, the Small Animal Teaching Hospital, School of Veterinary Medicine, were sampled, thereby limiting the generalizability of the findings from the study. This hospital was chosen because it is the largest veterinary clinical facility for pets in Ghana and also because of time and resource constraints.

## 3 Results

### 3.1 Background Information

The sociodemographic characteristics of respondents were as follows: males 131 (63%), females 77 (37%); species brought: dogs 188 (90.4%) and cats 20 (9.6%). The age groups were as follows: less than 20 years (n = 2, 1.0%); 20–29 years (n = 50, 24.0%); 30–39 years (n = 73, 35.1%); 40–49 years (n = 51, 24.5%); 50–59 years (n = 21, 10.1%) and 60 and above years (n = 11, 5.3%). No significant differences were seen in overall satisfaction (satisfied vs. dissatisfied) on basis of gender of respondent (*p* = 0.39), species of animals brought (*p* = 0.60) or age group range of respondents (*p* = 0.87).

### 3.2 Dimensions

The results of the various analyses for the dimensions (tangibles, reliability, responsiveness, empathy and assurance) are presented in this section.

Table 1 provides the mean scores and RII for the parameters within the dimensions used.

**TABLE 1:** Descriptive statistics and relative importance indices for various dimensions.

Dimension	Parameter	Mean score (Max = 5)	SD	RII
	The hospital has modern equipment	3.92	0.47	0.78
Tangibles	The physical facilities look nice	3.65	0.57	0.73
	Employees are neat in appearance	4.00	0.24	0.80
	Posters/notices/directions look attractive and are well‐positioned	3.23	0.85	0.65
Reliability	When a client has a problem, employees show a sincere interest in solving it	4.00	0.28	0.80
	The hospital has staff that clients can rely on	4.00	0.26	0.80
	Employees tell clients exactly when services will be performed	3.89	0.50	0.78
Responsiveness	Employees give prompt service to clients and patients	4.00	0.27	0.80
	Employees are always willing to help clients and patients	4.00	0.26	0.80
	Employees are never too busy to respond to clients’ request	4.00	0.22	0.80
	The behaviour of employees gives clients confidence	4.00	0.26	0.80
	Clients feel safe when being attended to at the hospital	4.00	0.25	0.80
Assurance	Employees are consistently courteous to clients	4.00	0.26	0.80
	Employees have the knowledge to answer clients' questions	4.00	0.25	0.80
	The hospital gives clients and patients individual attention	4.00	0.25	0.80
	The hospital has employees who give clients and patients personal attention	4.01	0.25	0.80
Empathy	The hospital has the client's and patient's best interests at heart	4.00	0.26	0.80
	Employees understand the specific needs of their clients and patients	4.00	0.25	0.80

Abbreviations: RII, Relative Importance Index; SD, standard deviation.

The Unweighted SERVPERF was calculated to be 818.6, while the Average Weighted SERVPERF was 3.9 (out of 5).

Table 2 presents the mean scores, Cronbach's alpha values and the RII for the dimensions.

**TABLE 2:** Descriptive statistics, reliabilities and RII.

Dimensions	Mean score	SD	Cronbach's alpha	RII scores
Tangibles	3.70	0.65	0.537	0.74
Reliability	4.00	0.27	0.846	0.80
Responsiveness	3.97	0.34	0.783	0.79
Assurance	4.00	0.25	0.984	0.80
Empathy	4.00	0.25	0.977	0.80

Abbreviations: RII, Relative Importance Index; SD, standard deviation.

Table 3 presents the model summary after stepwise regression analyses

**TABLE 3:** Model summary.

		Model summary			
Model	R	Predictor/outcome variable	R square	Adjusted R square	Std. error of the estimate
1	0.189	(Constant), Reliability average perception score and overall satisfaction	0.036	0.031	0.602
2	0.312	(Constant), Reliability average perception score, empathy average perception score and overall satisfaction	0.097	0.089	0.584

The ANOVA results for the models generated are shown in Appendix 1.

Table 4 shows the coefficients for the model equation.

**TABLE 4:** Coefficients for model equation.

		Unstandardized coefficients		Standardized coefficients	*t*	Significance	95.0% Confidence interval for B		Correlations			Collinearity statistics	
Model		*B*	Std. error	Beta			Lower bound	Upper bound	Zero‐order	Partial	Part	Tolerance	VIF
1	(Constant)	2.389	0.669		3.570	0.000	1.070	3.709					
	Reliability average perception score	0.462	0.167	0.189	2.764	0.006	.132	0.791	0.189	0.189	0.189	1.000	1.000
2	(Constant)	2.880	0.662		4.348	0.000	1.574	4.185					
	Reliability average perception score	2.443	0.554	1.001	4.412	0.000	1.351	3.534	0.189	0.294	0.293	0.086	11.683
	Empathy average perception score	−2.102	0.562	−0.849	−3.742	0.000	−3.210	−0.995	0.108	−0.253	−0.248	0.086	11.683

^a^ Dependent variable: Overall satisfaction.

The model equations generated were as follows:

**Model 1**
Overall satisfaction (mean score) = 2.39 + 0.19 × Reliability mean score.
**Model 2**
Overall satisfaction (mean score) = 2.88 + 1.00 × Reliability mean score–0.85 × Empathy mean score.

The second model had a variance inflation factor (VIF) of 11.68 for both reliability and empathy (Appendix 2), indicating high collinearity, so one had to be dropped.

Appendix 2 provides the collinearity diagnostics for the two models. The correlations between the various dimensions are shown in Appendix 3.

### 3.3 Indicators

The results of the various analyses involving the 15 indicators are presented in this section.

Table 5 gives the various proportions for the categories stated.

**TABLE 5:** Proportions of respondents according to categories of response.

	Very poor (1) *n* (%)	Poor (2) *n* (%)	Fair (3) *n* (%)	Good (4) *n* (%)	Very good (5) *n* (%)	No idea *n* (%)
Effectiveness	1 (0.5)	1 (0.5)	1 (0.5)	121 (58.2)	84 (40.4)	
Efficiency	0	1 (0.5)	0	126 (60.6)	81 (38.9)	
Service quality	0	0	1 (0.5)	112 (53.8)	95 (47.5)	
Staff attitude	0	0	1 (0.5)	103 (49.5)	104 (50.0)	
Staff competence	0	0	0	107 (51.4)	99 (47.6)	2 (1.0)

Availability of:	Unavailable (1) *n* (%)	Sometimes (2) *n* (%)	Available (3) *n* (%)	Always (4) *n* (%)		
Drugs	1 (0.5)	42 (20.2)	153 (73.6)	12 (9.8)		
Vaccinations	1 (0.5)	19 (9.1)	167 (80.3)	21 (10.1)		
Services	0	15 (7.2)	174 (84.1)	18 (8.7)		

	Unaffordable (1) *n* (%)	Fairly (2) *n* (%)	Affordable (3) *n* (%)			
Service affordability	1 (0.5)	33 (15.9)	174 (83.6)			

	Very expensive (1) *n* (%)	Expensive (2) *n* (%)	Reasonable (3) *n* (%)	Fair (4) *n* (%)		
Service charges	1 (0.5)	34 (16.3)	158 (76.0)	15 (7.2)		
Drug costs	0	34 (16.3)	157 (75.5)	14 (6.7)		3 (1.4)

	Too long (1) *n* (%)	Long (2) *n* (%)	Acceptable (3) *n* (%)	Short (4) *n* (%)		
Timeliness	2 (1.0)	5 (2.4)	191 (91.6)	10 (4.8)		

	Too long (1) *n* (%)	Long (2) *n* (%)	Reasonable (3) *n* (%)	Fair (4) *n* (%)		
Time spent	1 (0.5)	7 (3.4)	188 (90.4)	12 (5.8)		

	Very difficult (1) *n* (%)	Difficult (2) *n* (%)	Fair (3) *n* (%)	Easy (4) *n* (%)	Very easy (5) *n* (%)	
Service accessibility	0	1 (0.5)	3 (1.4)	121 (58.2)	83 (39.9)	

	Unaffordable (1) *n* (%)	Fairly (2) *n* (%)	Affordable (3) *n* (%)			
Service affordability	1 (0.5)	33 (15.9)	174 (83.6)			

	Poor (1) *n* (%)	Somehow (2) *n* (%)	Fairly (3) *n* (%)	Met (4) *n* (%)	Very much (5) *n* (%)	
Needs met	0	3 (1.4)	6 (2.9)	117 (85.1)	22 (10.6)	

	Very dissatisfied (1) *n* (%)	Dissatisfied (2) *n* (%)	Somehow satisfied (3) *n* (%)	Satisfied (4) *n* (%)	Very satisfied (5) *n* (%)	
Overall satisfaction	1 (0.5)	0	4 (1.9)	151 (72.6)	52 (25.0)	

Table 6 gives the mean scores and RII for indicators evaluated.

**TABLE 6:** Descriptive statistics and RII for indicators used (from highest to lowest RII).

	Mean score	SD	Maximum score	RII
Service affordability	2.83	0.39	3	0.94
Staff attitude	4.50	0.51	5	0.90
Service quality	4.45	0.51	5	0.89
Staff competence	4.44	0.66	5	0.89
Efficiency	4.38	0.52	5	0.88
Effectiveness	4.38	0.58	5	0.88
Service accessibility	4.38	0.54	5	0.88
Overall satisfaction	4.22	0.52	5	0.84
Meeting needs	4.05	0.44	5	0.81
Timeliness	3.05	0.50	4	0.76
Total time spent	3.01	0.33	4	0.75
Service availability	3.01	0.40	4	0.75
Vaccinations availability	3.00	0.46	4	0.75
Service charges	2.90	0.49	4	0.73
Drugs availability	2.89	0.63	4	0.72
Drug costs	2.86	0.59	4	0.72

Abbreviations: RII, Relative Importance Index; SD, standard deviation.

Definitions.

• Service affordability: Ability to pay for services.

• Staff attitude: Interpersonal relations shown by staff.

• Service quality: The degree to which service meets the client's expectations.

• Staff competence: Technical competence of staff (Knowledge, skills, and actual performance).

• Efficiency: How well are resources used to achieve desirable results?

• Effectiveness: How effective do you think the hospital is in reducing discomfort, disease, death and dissatisfaction?

• Service accessibility: How easy are individuals able to reach and obtain services?

• Overall satisfaction with services received.

• Meeting needs: Meeting the needs of clients for veterinary services.

• Timeliness: Time taken to receive help.

• Total time spent: Time from arrival to leaving the facility.

• Service availability: Services are provided when needed.

• Vaccinations availability: Availability of pet vaccinations.

• Service charges: Charges for services rendered.

• Drugs availability: Availability of veterinary drugs in hospital for sale to clients.

• Drug cost: Cost of drugs on the market.

Table 7 provides values for comparison between satisfied and dissatisfied respondents using the various indicators.

**TABLE 7:** Statistical values comparing satisfied and dissatisfied groups for various indicators tested.

Indicator	Category	Satisfied		Dissatisfied		Fisher	Spearman		Mantel–Haenzel	
		*n*	%	*n*	%	*p*	Value	*p*	*χ* ^2^	*p*
Effectiveness	Poor	0	0	1	0.5	0.024*	0.443	0.000*	9.656	0.002*
	Good	203	97.6	4	2.0					
Efficiency	Poor	0	0	1	0.5	0.024*	0.443	0.000*	9.656	0.002*
	Good	203	97.6	4	2.0					
Service accessibility	Poor	1	0.5	0	0	0.976	−0.011	0.876	9.656	0.002*
	Good	202	97.1	5	2.4					
Service quality	Poor	5	2.4	0	0	nd	nd	nd	nd	nd
	Good	203	97.6	0	0					
Staff attitude	Poor	0	0	0	0	nd	nd	nd	nd	nd
	Good	203	97.6	5	2.4					
Staff competence	Poor	1	0.5	0	0	1.000	−0.011	0.876	9.656	0.002*
	Good	202	97.1	5	2.4					
Timeliness	Unacceptable	5	2.4	1	0.5	0.137	0.160	0.021*	0.921	0.337
	Acceptable	198	95.2	4	2.0					
Service charges	Expensive	35	16.8	0	0	0.592	−0.071	0.311	0.170	0.680
	Reasonable	168	80.8	5	2.4					
Drugs availability	Unavailable	40	19.2	3	1.4	0.061	0.152	0.028*	2.674	0.102
	Available	163	78.4	2	1.0					
Drug cost	Expensive	37	17.8	0	0	0.588	−0.073	0.295	0.211	0.646
	Reasonable	166	79.8	5	2.4					
Vaccinations availability	Unavailable	18	8.7	2	1.0	0.074	0.162	0.020*	2.438	0.118
	Available	185	88.9	3	1.4					
Service affordability	Unaffordable	1	0.5	0	0	1.000	−0.011	0.876	9.656	0.002*
	Affordable	202	97.1	5	2.4					
Service availability	Unavailable	14	6.7	1	0.5	0.315	0.078	0.265	0.059	0.808
	Available	189	90.9	4	1.9					
Needs met	Unmet	2	1.0	1	0.5	0.071	0.244	0.000*	2.627	0.105
	Met	201	99.6	4	1.9					
Time spent	Too long/long	6	2.9	2	1.0	0.012*	0.25	0.000*	9.430	0.002*
	Fair	197	94.7	3	1.4					

Abbreviation: nd, not determined.

* Significant at *p* < 0.05.

The model summary was as follows: Cox and R square was 0.119 and Nagelkerke R Square was 0.584.

Table 8 provides information on logistic regression modelling done from the data collected.

**TABLE 8:** Variables in the model equation.

		*B*	S.E.	Wald	*df*	Significance	Exp(B)	95% C.I. for EXP(B)	
								Lower	Upper
Step 1^a^	Gender (1)	−17.474	4016.470	0.000	1	0.997	0.000	0.000	–
	EffectP (1)	43.803	55,671.428	0.000	1	0.999	10,548,421,362,748,652,000.000	0.000	
	EfficP (1)	109.568	60,307.355	0.000	1	0.999	3.842E+47	0.000	–
	AccessP (1)	−16.969	40,192.962	0.000	1	1.000	0.000	0.000	–
	CompetenceP (1)	−3.893	40,515.843	0.000	1	1.000	0.020	0.000	–
	TimelyP (1)	−14.652	41,120.409	0.000	1	1.000	0.000	0.000	–
	ChargesP (1)	−15.971	5285.009	0.000	1	0.998	0.000	0.000	–
	DrugsAvailP (1)	2.730	1.275	4.585	1	0.032	15.333	1.260	186.600
	DrugCostP (1)	−15.806	5104.718	0.000	1	0.998	0.000	0.000	–
	VaccinationP (1)	−17.167	7538.632	0.000	1	0.998	0.000	0.000	–
	AffordP (1)	31.777	57,314.398	0.000	1	1.000	63,208,488,988,597.520	0.000	–
	ServicesP (1)	−16.671	8317.311	0.000	1	0.998	0.000	0.000	–
	NeedsP (1)	−19.699	40,192.977	0.000	1	1.000	0.000	0.000	–
	TimeSpentP (1)	−1.198	37,775.674	0.000	1	1.000	0.302	0.000	–
	Constant	−44.144	105,635.620	0.000	1	1.000	0.000		

^a^ Variable(s) entered on step 1: Gender, EffectP, EfficP, AccessP, CompetenceP, TimelyP, ChargesP, DrugsAvailP, DrugCostP, VaccinationP, AffordP, ServicesP, NeedsP and TimeSpentP.

The prediction model equation was as follows: Overall satisfaction = −44.1 + 15.3 × Drugs availability.

### 3.4 Client Satisfaction Calculations

The CSAT was calculated as ((151 + 52)/208) × 100) to give 97.5%. The mean satisfaction rate (calculated as stated in Section 2) was 84.3%. The Composite Customer Satisfaction Score calculated as ((877/(5×208)) ×100) was also 84.3%. The Customer Satisfaction Index was calculated to be 81.5%.

## 4 Discussion

The major species seen at the hospital was dogs (90%), supporting an earlier finding (Turkson 2023). There were no differences in respondents’ satisfaction on the basis of gender, age group of respondents or the pet species kept, suggesting that these may have no influence on client satisfaction with delivery of pet services.

The Cronbach alpha value of 0.8 obtained for the dimensions portion (Table 2) was above the threshold of 0.6 (Nagpal et al. 2010), indicating that the questionnaire was reliable. The lowest Cronbach's alpha value was for tangibles (0.537) and the highest was for assurance (0.984) (Table 2). In contrast, the lowest alpha value in a study in Sri Lanka was for assurance, while the highest was for empathy (Dassanayake and Weerasiri 2015). Apart from tangibles, all the other alpha values were above 0.7 (Table 2), which is the threshold value for internal consistency (Dassanayake and Weerasiri 2015).

Our results showed that tangibles had the lowest perception mean score (3.7), compared to the other factors (Table 2). Tangibles have been identified as one factor lacking in developing countries (Byju and Srinivasulu 2014), requiring improvement. Generally, the respondents in this study were satisfied with the other factors of service quality. In a Sri Lankan veterinary hospital, the most important dimension for customer satisfaction was empathy, followed by responsiveness and assurance (Dassanayake and Weerasiri 2015). In our study, reliability, assurance and empathy had the highest RII (0.80), with tangibles having the least value (0.74) (Table 2). According to Ali et al. (2021), customers perceive reliability as the dominant service quality and suggested that improvements here could lead to customer loyalty. Our study found reliability to be the only dimension in the prediction equation generated. Reliability is reported to have a direct positive effect on customer satisfaction (Ngaliman, Mika Giofani Eka, and Suharto 2019) and could create satisfaction for customers (Ahmed et al. 2017; Rehman 2012). Responsiveness, empathy and tangibility had a significant impact on customer satisfaction in the health sector in India (Byju and Srinivasulu 2014).

A regression model for overall satisfaction used only the reliability average perception score with tolerance and VIF both being 1 (Table 4). The reliability score explained 46% of the total variance in overall satisfaction. The regression analysis suggested that the most important service dimension for significant changes in customer satisfaction was reliability. A second regression model had reliability average perception score and empathy average perception score with tolerance and VIF being 0.086 and 11.683, respectively (Table 4). Tolerance and VIF values are used to check for multicollinearity (Dassanayake and Weerasiri 2015). Both reliability and empathy had VIF value above 5 (the threshold), indicating that the variables were highly correlated (Independent Media Associates Inc., 2024). In such cases, the model's predictive power is not reduced, but the coefficients may not be significant statistically with a Type II error (CFI, 2024). Where VIF is above 10 or tolerance is lower than 0.1, there is significant multicollinearity that needs correction (CFI, 2024). The independent variables had tolerance values lower than the threshold value, and VIF was also above the threshold of 10. These provided ample evidence of multicollinearity, leading to the rejection of the second model.

Table 5 shows a trend where the positive portions of the scales used had dominant proportions implying a positive skew. Table 6 shows service affordability as the indicator with the highest RII (0.94), with drug costs and availability having the lowest, pointing to indicators that should be prioritized and targeted for client satisfaction.

Significant differences were seen in the proportions for satisfied and dissatisfied groups for effectiveness, efficiency and time spent in the Fisher, Spearman and Mantel–Haenzel tests (Table 7). In addition, there were significant differences in proportions for timeliness, drugs availability, pet vaccinations availability and meeting the needs of clients in the two groups in the Spearman test (Table 7). In the Mantel–Haenzel test, additional differences were seen in service accessibility, staff competence and service affordability (Table 7). These may serve as pointers to areas for improvement in client satisfaction. Customer satisfaction produces a positive attitude, resulting in a greater likelihood of further use (White 2010).

There were very high and significant correlations between the 15 indicators, implying strong multicollinearity. The model correctly classified 98.6% of cases overall. The regression model had only drug availability as the independent variable. It was difficult to assign reasons for this finding. The omnibus test of model coefficient had alpha value of 0.024, implying the model was significant. Likewise, the Cox and Snell R square and the Nagelkerke R square values suggested that between 11.9% and 58.4% of the variance in the dependent variable was explained by the model.

The client satisfaction indices calculated in this study were very high (84%–98%), which is highly commendable. A CSI score of 80% and above is said to be good (Process Street n.d.). Factors affecting CSI include quality of the service, customer service, price and value and brand reputation (Process Street n.d.). The hospital should take these into consideration, making every effort to improve and maintain these scores.

## 5 Conclusion

The study provides attributes considered by clients as contributing to satisfaction with veterinary services delivery for pets in Accra, Ghana. Service affordability had the highest RII. Client satisfaction was very high. Regression analyses revealed reliability mean score for dimensions and availability of drugs for indicators as predictors of overall satisfaction with services delivery. Future research directions should replicate the study in other veterinary facilities in Ghana and elsewhere in Africa, since this is the first published study of its kind, as far as the authors are aware.

## Author Contributions

Paa Kobina Turkson: Conception, formulation and generation of data collection tool, data analyses and interpretation, manuscript writeup and review. Celine Naa Dede Coppson: Data collection, input and analyses, manuscript review. Anthony Joe Turkson: Data analyses and manuscript review.

## Ethics Statement

The authors confirm that the ethical policies of the journal as noted on the journal's author guidelines page have been adhered to. No ethical approval was required since the data collected from clients did not have any personal identifiers ensuring anonymity. Approval for the work was given by the coordinator of the companion animal hospital used.

## Conflicts of Interest

The authors declare no conflicts of interest.

### Peer Review

The peer review history for this article is available at https://publons.com/publon/10.1002/vms3.70331.

## Data Availability

The data are available from the first author upon reasonable request.

## Appendix 1 ANOVA Results

### 1.1

**Table:**

ANOVA^a^						
Model		Sum of squares	*df*	Mean square	*F*	Significance
1	Regression	2.769	1	2.769	7.638	0.006^b^
	Residual	74.688	206	0.363		
	Total	77.457	207			
2	Regression	7.545	2	3.772	11.062	0.000^c^
	Residual	69.912	205	0.341		
	Total	77.457	207			

^a^ Dependent variable: Overall satisfaction.

^b^ Predictors: (Constant), Reliability Average perception score.

^c^ Predictors: (Constant), Reliability Average perception score, Empathy Average perception score.

## Appendix 2 Collinearity Diagnostics

### 2.1

**Table:**

Collinearity diagnostics^a^						
Model	Dimension	Eigenvalue	Condition index	Variance proportions		
				(Constant)	Reliability Average perception score	Empathy Average perception score
1	1	1.998	1.000	0.00	.00	
	2	0.002	32.031	1.00	1.00	
2	1	2.997	1.000	0.00	0.00	0.00
	2	0.003	34.613	1.00	0.02	0.02
	3	0.000	133.716	0.00	0.98	0.98

^a^ Dependent Variable: Overall satisfaction.

## Appendix 3 Correlation Values for Average Scores of Various Dimensions

### 3.1

**Table:**

	Spearman rho	
	Value	Significance
Overall satisfaction × Tangibles	0.020	0.769
Overall satisfaction × Reliability	0.217	0.000^*^
Overall satisfaction × Responsiveness	0.103	0.137
Overall satisfaction × Assurance	0.174	0.012^*^
Overall satisfaction × Empathy	0.173	0.012^*^
Tangibles × Reliability	0.045	0.515
Tangibles × Responsiveness	0.151	0.030^*^
Tangibles × Assurance	0.097	0.162
Tangibles × Empathy	0.096	0.170
Reliability × Responsiveness	0.494	0.000^*^
Reliability × Assurance	0.805	0.000^*^
Reliability × Empathy	0.804	0.000^*^
Responsiveness × Assurance	0.535	0.000^*^
Responsiveness × Empathy	0.535	0.000^*^
Assurance × Empathy	1.000	0.000^*^

* Significant at *p* < 0.05.
